# Molecular Barcoding of Aquatic Oligochaetes: Implications for Biomonitoring

**DOI:** 10.1371/journal.pone.0125485

**Published:** 2015-04-09

**Authors:** Régis Vivien, Sofia Wyler, Michel Lafont, Jan Pawlowski

**Affiliations:** 1 Department of Genetics and Evolution, University of Geneva, Geneva, Switzerland; 2 Self-entrepreneur, Villeurbanne, France; University of Veterinary Medicine Hanover, GERMANY

## Abstract

Aquatic oligochaetes are well recognized bioindicators of quality of sediments and water in watercourses and lakes. However, the difficult taxonomic determination based on morphological features compromises their more common use in eco-diagnostic analyses. To overcome this limitation, we investigated molecular barcodes as identification tool for broad range of taxa of aquatic oligochaetes. We report 185 COI and 52 ITS2 rDNA sequences for specimens collected in Switzerland and belonging to the families Naididae, Lumbriculidae, Enchytraeidae and Lumbricidae. Phylogenetic analyses allowed distinguishing 41 lineages separated by more than 10 % divergence in COI sequences. The lineage distinction was confirmed by Automatic Barcode Gap Discovery (ABGD) method and by ITS2 data. Our results showed that morphological identification underestimates the oligochaete diversity. Only 26 of the lineages could be assigned to morphospecies, of which seven were sequenced for the first time. Several cryptic species were detected within common morphospecies. Many juvenile specimens that could not be assigned morphologically have found their home after genetic analysis. Our study showed that COI barcodes performed very well as species identifiers in aquatic oligochaetes. Their easy amplification and good taxonomic resolution might help promoting aquatic oligochaetes as bioindicators for next generation environmental DNA biomonitoring of aquatic ecosystems.

## Introduction

Oligochaetes are an important group of freshwater benthic invertebrates, abundant in fine, sandy and coarse sediments of watercourses and lakes, as well as in the hyporheic zone and groundwater [1, 2]. The group includes a large number of species encompassing a wide range of pollution sensitivity [3]. Oligochaetes are used to assess the quality of aquatic ecosystem in field and laboratory studies, through different approaches: study of their community composition and structure, ecotoxicological tests and bioaccumulation studies [4].

Since 1960, oligochaetes have been used in many countries for assessing the ecological quality of watercourses and lakes [5–10]. Three methods of bioindication based on the analysis of sediment-dwelling oligochaete assemblages have been developed: the Oligochaete Index of Sediment Bioindication (IOBS) to assess the quality of fine / sandy sediments of watercourses [11, 12], the Oligochaete Index of Lake Bioindication (IOBL) to assess the quality of fine sediments of lakes [13–15] and the Functional Traits (FTrs) method to assess the ecological effects of interactions between physical and chemical factors, and particularly the ecological effects of the dynamics of water exchanges between groundwater and surface water [1, 15, 16]. The IOBS index has been applied for some years by the Water Ecology Service of the canton of Geneva (Switzerland) as part of watercourses quality monitoring program [2, 17]. Moreover, oligochaetes have been used for decades in Switzerland to assess the ecological quality of lake sediments [18–20].

Aquatic oligochaete main families in Europe are Naididae (comprising Naidinae, Tubificinae, Rhyacodrilinae and other subfamilies), Enchytraeidae, Lumbriculidae, Haplotaxidae and Propappidae [4]. The earthworm family Lumbricidae family also includes some aquatic or amphibious forms. Morphological identification of oligochaete species is not easy. Most species of Tubificinae, Lumbriculidae and Enchytraeidae cannot be identified in an immature state. These unidentifiable specimens can represent over 80% of specimens of a single sample. In addition, the identification of the vast majority of Lumbriculidae and Enchytraeidae species is difficult and requires practicing dissection, which is unrealizable in routine analyses. Furthermore, various molecular studies have revealed the existence of cryptic species within aquatic oligochaetes [21–25].

DNA barcoding allows for the rapid identification of a species by matching the sequence of a short fragment of a selected gene to a reference library containing DNA sequences generated from identified specimens [26, 27]. Many studies show that mitochondrial COI gene is a very effective barcode for identification of aquatic and terrestrial oligochaetes [4, 25, 28–30]. COI barcodes have already been established for several species of freshwater aquatic oligochaetes but their taxonomic range and geographic origin of sequenced specimens was relatively restricted [21, 24, 25, 31].

The development of molecular barcoding and its application in the field of oligochaete indices would allow to identify all the species present in a sample and thus to make results more reliable and more accurate. However, the routine use of DNA barcodes requires the estimation of species richness that depends on genetic divergence threshold values used for species identification. Species boundaries delimitation is subject to controversy and distance thresholds seem to be taxon specific [32]. Erséus and Gustafsson [33] and Zhou et al. [34] suggested a 10% threshold of COI divergence for segregating between congeneric species in aquatic oligochaetes. Achurra & Erséus [27] showed that COI barcode analysis were not sufficient to delimit the oligochaete species *Stylodrilus heringianus* Claparède, 1862 and suggested to combine COI analysis with nuclear data. The ITS region has been proposed as a complementary marker to COI for aquatic oligochaetes [21, 27].

In the present study, we tested the efficiency of COI as oligochaete barcode for discriminating species in biomonitoring studies by comparing the taxonomic diversity obtained with genetic analysis with that obtained with the classical morphological approach. In addition, we sequenced the ITS2 region of most of the lineages in order to validate species boundaries delimitations established based on COI data. We found that the diversity of oligochaetes is underestimated by morphological approach and that the COI sequences, at 10% divergence threshold, accurately identify most of the lineages.

## Material and methods

### Sampling and morphological analysis

Oligochaetes were sampled between October 2012 and July 2013 in eight watercourses of the canton of Geneva (Switzerland) and in two streams of the canton of Vaud (Switzerland) (S1 Table). The Water Ecology Service of the State of Geneva issued the permission to conduct this study on these sites. Fine and/or sandy sediment were sieved in the laboratory (0.2 mm sieve mesh size) and live oligochaetes were sorted out. The worms were cut in two. The anterior parts were fixed and preserved in formalin 5% or in 100% alcohol and the posterior parts were preserved frozen (-20°C), some being fixed in 100% alcohol. Anterior parts were cleared in an acid lactic / glycerol solution and mounted between slide and coverslip in a permanent coating solution composed of lactic acid, glycerol and polyvinylic alcohol (Mowiol 4–88). These anterior parts served as reference vouchers and have been deposited at the Museum of Natural History of the city of Geneva. Oligochaete specimens were identified at the level of species, genus and family by using the following reference bibliography: Sperber [35], Brinkhurst [36], Timm & Veldhuijzen van Zanten [37], Erséus et al. [38], Timm [39], Rodriguez & Achurra [40]. In the case of unrecognizable immature Tubificinae, the specimens were assigned to a group with or without hair setae.

### Genetic analyses

Total genomic DNA was extracted using guanidine thiocyanate as described by Tkach & Pawlowski [41]. COI gene was amplified using LCO 1490 and HCO 2198 primers [42], while ITS2 rRNA gene was amplified using the primers described in [43]. PCRs were performed in 20μL total volume with 0.60U Taq (Roche), 2μL of the 10X buffer containing 20mM MgCl2, 0.8μL of each primer (10mM), 0.4 μl of a mix containing 10mM of each dNTP (Roche) and 0.8μL template DNA of unknown concentration. The PCR program comprised an initial denaturation at 95°C for 5 min, followed by 35 cycles of 95°C for 40 s, annealing at 44°C for 45 s and 72°C for 1 min, with a final elongation step at 72°C for 8 min. COI PCR products were then directly and bi-directionally sequenced on an ABI 3031 automated sequencer (Applied Biosystems) using the same primers and following the manufacturer’s protocol. Some of the ITS2 sequences were obtained by direct bi-directional sequencing, but most of the ITS2 PCR products were cloned with the TOPO10 kit from Invitrogen. Between one and four clones per specimen were sequenced.

Sequence editing and generation of consensus sequences were accomplished using CodonCode Aligner (CodonCode Corporation). Alignments were automatically generated using Muscle [44] as implemented in Seaview program [45] and verified manually. Phylogenetic trees were reconstructed using PhyML [46] or NJ as implemented in Seaview [45], with 100 and 1000 bootstrap replicates, respectively. The illustrated trees were constructed with PhyML. Only the bootstrap values (BV) higher than 80% are shown on the trees. We used GTR + G + I model as indicated by MEGA5 [47].

Our COI sequences were compared to Genbank (NCBI) sequences using BLAST (www.ncbi.nlm.nih.gov/BLAST/Blast.cgi). Only very similar sequences from Genbank were retained (98% or more). In total, twenty-two COI sequences were added to our dataset.

Intra and inter-lineage genetic distance (COI and ITS2) calculations were based on K2P model, as implemented in MEGA 5.1 software [47].

On the illustrated COI tree, the sequences that were identical or diverged by less than 1% were combined, but all sequences of the dataset were taken into account for the genetic distance calculations.

A median joining network of the ITS2 sequences was drawn using the software Network [48]. Both site mutations and indels were equally weighted and all the structural mutations (insertions/deletions of motifs of more than 1 bp and the inversion) were treated as single-step events.

The Automatic Barcode Gap Discovery (ABGD) method for species delimitation [49] was applied in order to validate species delimitations based on COI distances. The defaults values were used as given at ABGD website http://wwwabi.snv.jussieu.fr/public/abgd/abgdweb.html, except for the relative gap width, which was set at 1.0.

The sequences are accessible in the European Nucleotide Archive (ENA) at http://www.ebi.ac.uk/ena/data/view/LN810242-LN810426 for COI and at http://www.ebi.ac.uk/ena/data/view/LN810169-LN810241 for ITS2. The accession numbers are provided as S2 Table.

## Results

### COI analysis

A total of 185 specimens belonging to four families were sequenced (Table 1), including 154 Naididae (80 Tubificinae with hair setae, 49 Tubificinae without hair setae, 16 Naidinae and 3 Rhycodrilinae), 26 Lumbriculidae, 5 Enchytraeidae and 6 Lumbricidae. The number of sequences per lineage varies from 1 to 44.

**Table 1:** List of the lineages of aquatic oligochaetes.

	Lineage No	COI sequences	ITS2 specimens / sequences (*)	Morphologically identified specimens	COI intra-lineage variability (%)	COI inter-lineage variability (%)	ITS2 intra-lineage variability (%)	ITS2 inter-lineage variability (%)
**Naididae**								
**Tubificinae**								
Tub. with hair setae	T1	1	1 / 1 (3)	1	NC	18.935	NC	5.274
Tub. with hair setae	T2	6	1 / 1 (3)	6	0.000–0.160	17.068	NC	20.870
Tub. with hair setae	T3	1	1 / 1 (1)	1	NC	17.207	NC	5.537
*Aulodrilus pluriseta* (Piguet, 1906)	T4	1	1 / 1 (4)	1	NC	23.893	NC	> 20
*Branchiura sowerbyi* Beddard, 1892	T5	5	1 / 1 (4)	5	0.000	23.121	NC	> 20
*Lophochaeta ignota* Stolc, 1886	T6	1	1 / 1 (4)	1	1.706	19.142	NC	20.870
*Potamothrix bavaricus* (Oschmann, 1913)	T7	44	2 / 3 (3)	19	0.000–0.723	18.528	0.000	4.837
*Psammoryctides barbatus* (Grube, 1861)	T8	4	1 / 1 (1)	4	0.000–1.694	20.588	NC	22.234
*Tubifex tubifex* (Müller, 1774)	T9	4	1 / 2 (5)	2	0.000–1.826	20.891	0.000	1.672
*Tubifex tubifex* (Müller, 1774)	T10	4	2 / 2 (8)	3	0.000–9.966	21.784	0.000	1.672
*Tubifex tubifex* (Müller, 1774)	T11	5	2 / 2 (7)	1	0.000–2.841	24.589	0.000	3.897
*Tubifex tubifex* (Müller, 1774)	T12	3	1 / 3 (3)	1	0.000–2.566	24.589	0.465–0.935	3.897
*Tubifex montanus* Kowalewski, 1919	T13	1		1	NC	23.995	NT	NT
Tub. without hair setae	T14	1	1 / 1 (4)	1	NC	18.548	0.000	3.568
Tub. without hair setae	T15	3	3 / 3 (12)	3	0.163–1.067	18.548		
Tub. without hair setae	T16	1	1 / 2 (5)	1	NC	20.541	0–0.714	2.113
*Limnodrilus hoffmeisteri* Claparede, 1862	T17	22	2 / 6 (9)	12	0.000–9.188	20.801		
*Limnodrilus hoffmeisteri* Claparede, 1862	T18	5	4 / 5 (16)	3	0.000–2.257	19.820	0–0.597	2.113
*Limnodrilus hoffmeisteri* Claparede, 1862	T19	1	1 / 2 (2)	1	NC	19.084	0.471	4.131
*Limnodrilus hoffmeisteri* Claparede, 1862	T20	5	1 / 1 (1)	2	0.000	17.281	NC	4.131
*Limnodrilus hoffmeisteri* Claparede, 1862	T21	5		3	0.000–0.951	18.672	NT	NT
*Limnodrilus claparedianus* Ratzel, 1868	T22	5	3 / 8 (8)	1	0.203–5.040	17.731	0.000–2.116	4.379
*Limnodrilus udekemianus* Claparede, 1862	T23	1	1 / 2 (2)	1	NC	18.627	0.000	15.785
**Naidinae**								
*Chaetogaster diaphanus* (Gruithuisen, 1828)	N1	1	1 / 1 (4)	1	1.074	16.709	NC	23.74
*Nais bretscheri* Michaelsen, 1899	N2	1	1 / 1 (3)	1	0.312	16.709	NC	17.434
*Nais communis* Piguet, 1906	N3	1	1 / 1 (1)	1	0.000	12.608	NC	17.434
*Nais elinguis* Müller, 1774	N4	9	1 / 1 (1)	9	0.000–1.693	15.490	NC	14.129
*Ophidonais serpentina* (Müller, 1774)	N5	1	1 / 1 (1)	1	0.000	15.490	NC	15.943
*Piguetiella blanci* (Piguet, 1906)	N6	3	1 / 1 (1)	3	0.000	12.608	NC	14.129
**Rhyacodrilinae**								
*Bothrioneurum vejdovskyanum* Stolc, 1886	R1	3	2 / 2 (2)	3	0.305–5.109	25.929	0.585	35.809
**Lumbriculidae**								
Lumbriculidae unrecognizable in an immature state	LL1	2	1 / 1 (1)	2	0.305	26.045	NC	14.382
*Lumbriculus variegatus* (Muller, 1774)	LL2	2	2 / 3 (6)	2	0.152–1.458	20.509	0.419–0.840	15.927
*Stylodrilus heringianus* Claparède, 1862	LL3	22	2 / 2 (2)	7	0.000–6.863	23.866	0.474	14.382
**Enchytraeidae**								
*Enchytraeus buchholzi* Vejdovsky, 1878	E1	1	1 / 1 (4)	1	NC	19.640	NC	>20
*Fridericia* sp.	E2	1	1 / 2 (5)	1	NC	19.640	0.314	>20
*Lumbricillus rivalis* Levinsen, 1884	E3	2	1 / 1 (4)	2	0.000	22.500	NC	29.195
*Marionina argentea* (Michaelsen, 1889)	E4	1	1 / 1 (4)	1	NC	22.500	NC	29.195
**Lumbricidae**								
*Dendrodrilus rubidus* (Savigny, 1826)	LC1	1		0	2.852	18.432	NT	NT
*Eiseniella tetraedra* (Savigny, 1826)	LC2	2	1 / 1 (4)	2	0.164	10.157	NC	1.763
*Eiseniella tetraedra* (Savigny, 1826)	LC3	2	2 / 4 (5)	2	8.099	10.157	0.566–1.423	1.763
*Helodrilus oculatus* Hoffmeister, 1845	LC4	1		0	2.431	18.432	NT	NT

Lineages distinguished by 10% threshold in COI sequences, with data on number of COI sequences, number of ITS2 sequences (sequenced specimens, sequences represented in ITS2 tree, total sequences), number of morphologically identified specimens and the intra- and inter-lineage variability for COI and ITS2.

Tub. with hair setae = unidentified immature Tubificinae with hair setae; Tub. without hair setae = unidentified immature Tubificinae without hair setae; NT = not tested; NC = not calculated as the lineage contains only one sequence;

(*) = total number of sequences

**Fig 1:**
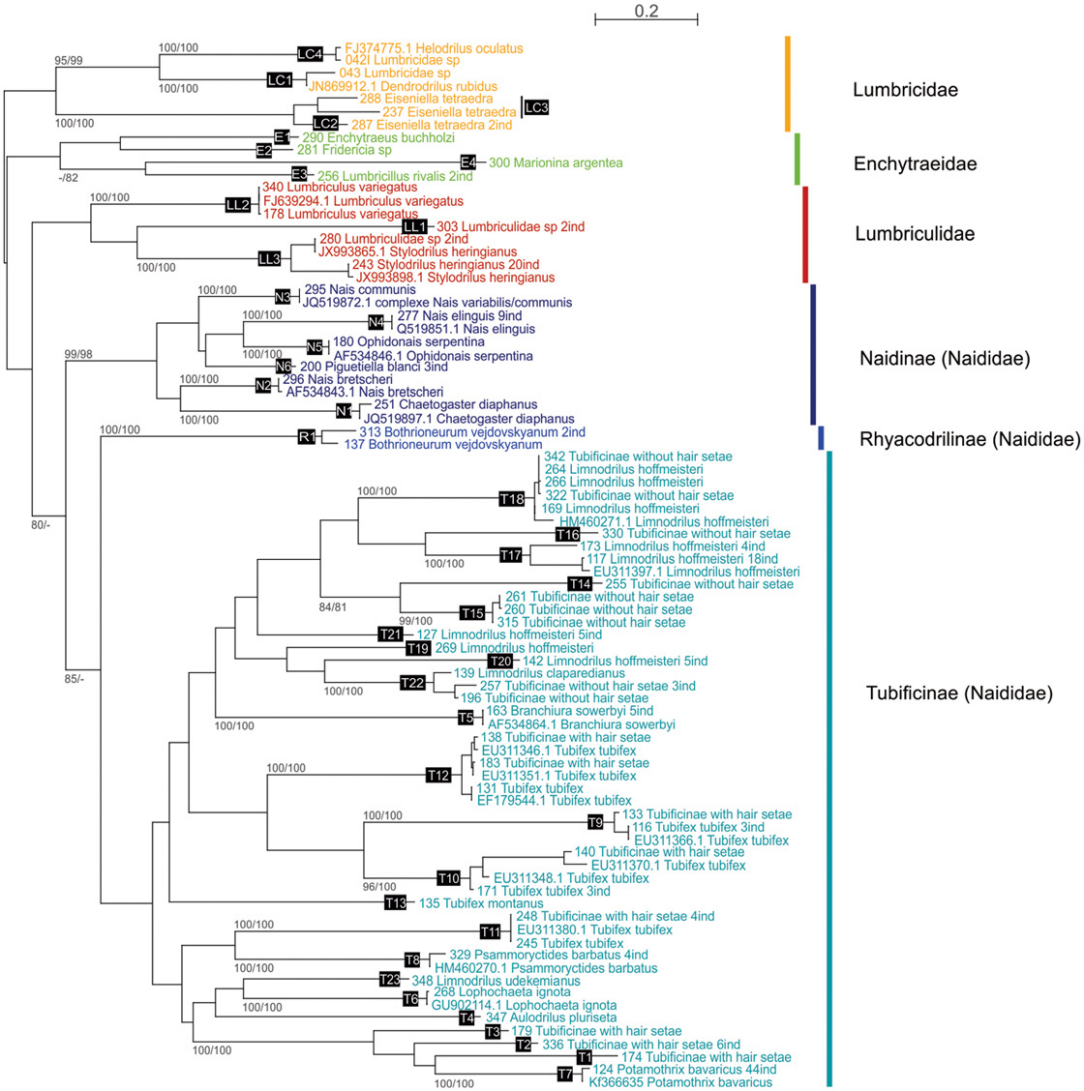
Maximum likelihood phylogenetic tree based on COI gene sequences. The numbers indicated at the black background represent the lineage numbers reported in Table 1. The numbers above the internal nodes correspond to bootstrap values of ML and NJ analyses; only those higher than 80% are indicated. The names of taxa comprise the number of isolate, the morphological identification and the accession number in the case of sequences from GenBank. When several sequences of less than 1% divergence were obtained for the same phylotype, their number is followed by "ind" placed after the names of the taxa.

The phylogenetic tree inferred from COI sequences (Fig 1) is in broad agreement with morphological classification. The three families Enchytraeidae, Lumbricidae and Lumbriculidae and the subfamilies Naidinae and Tubificinae are monophyletic. Only the subfamily Naidinae is well supported (BV ML = 0.99). Tubificinae with and without hair setae tend to group together, but the relations within this subfamily are not well resolved. The common genera *Tubifex* and *Limnodrilus* appear as non monophyletic, but there is low support for phylogeny of both genera.

Forty-one lineages are distinguished when considering the 10% threshold of genetic divergence. The application of the ABGD method confirms all these lineage delimitations, except for LC2 and LC3 that are regrouped. Among the 41 lineages, 26 can be associated to known species or genera. For seven out of these 26 lineages, no sequences existed in Genbank. These newly sequenced species are: *Tubifex montanus* Kowalewski, 1919, *Limnodrilus claparedianus* Ratzel, 1868, *Limnodrilus udekemianus* Claparède, 1862, *Piguetiella blanci* (Piguet, 1906), *Eiseniella tetraedra* (Savigny, 1826), *Bothrioneurum vejdovskyanum* Stolc, 1886 and *Fridericia* sp. The sequences of *Fridericia* spp., *Aulodrilus pluriseta* (Piguet, 1906), *Marionina argentea* (Michaelsen, 1889), *Lumbricillus rivalis* Levinsen, 1884 and *Enchytraeus buchholzi* Vejdovsky, 1878 present in Genbank are different from the sequences obtained in our study. The Genbank sequences of *E*. *bucholzi* and *L*. *rivalis* branch with our sequences of these species but the Genbank sequences of *A*. *pluriseta* and *M*. *argentea* do not branch with our sequences. In fact, Genbank sequence of *M*. *argentea* (GU 902092.1) branches with Lumbriculidae, so can not correspond to *M*. *argentea*. The lineages T1, T2 and T3 seem to belong to the genus *Potamothrix* as they all branch with *Potamothrix bavaricus*. The sequences of these lineages branch with the common species of *Potamothrix* (*P*. *hammoniensis*, *P*. *vejdovski*, *P*. *bedoti*, *P*. *moldaviensis* and *P*. *heuscheri*) present in Genbank but are different (more than 10% of divergence). Four lineages were attributed to *Tubifex tubifex* (Müller, 1774) (lineage T10), *Stylodrilus heringianus* (lineage LL3), *Helodrilus oculatus* Hoffmeisteri, 1845 and *Dendrodrilus rubidus* (Savigny, 1826) on the basis of GenBank data but their identification could not be verified, either because of their immature state or due to lack of expertise for terrestrial species.

Seven lineages are totally new. The specimens corresponding to these unidentified lineages are morphologically unrecognizable due to their immature state. Six of them belong to Tubificinae (lineages T1, T2, T3, T14, T15, T16) and one to Lumbriculidae (lineage LL1). This later lineage is represented by two specimens and is characterized by single-pointed chaetae; ventral chaetae in II, III and IV longer than dorsal chaetae; invisible double segmentation; prostomium rounded and flattened, almost as large as the segment I.

The remaining lineages correspond to cryptic species. Their number varies depending on selected threshold. When using the threshold of 10%, 11 cryptic species are identified: four in *Tubifex tubifex*, five in *Limnodrilus hoffmesteri* Claparède, 1862 and two in *Eiseniella tetraedra*. With a 5% threshold, four additional lineages can be distinguished within lineages T10 of *Tubifex tubifex*, T17 of *Limnodrilus hoffmeisteri*, LC3 of *Eiseniella tetraedra* and LL3 of *Stylodrilus heringianus*. These additional lineages diverge by 9.97, 9.2, 8.1 and 6.8%, respectively.

### ITS2 analysis

The ITS2 sequences were obtained for 37 out of the 41 lineages determined by COI analysis with the 10% threshold (Table 1). The species *Tubifex montanus*, *Dendrodrilus rubidus*, *Helodrilus oculatus* and the lineage T21 of *Limnodrilus hoffmeisteri* were not tested. One of the two sublineages T17 of *Limnodrilus hoffmeisteri* could not be analysed due to unsuccessful PCR amplification. At least 1 specimen per lineage was sequenced.

**Fig 2:**
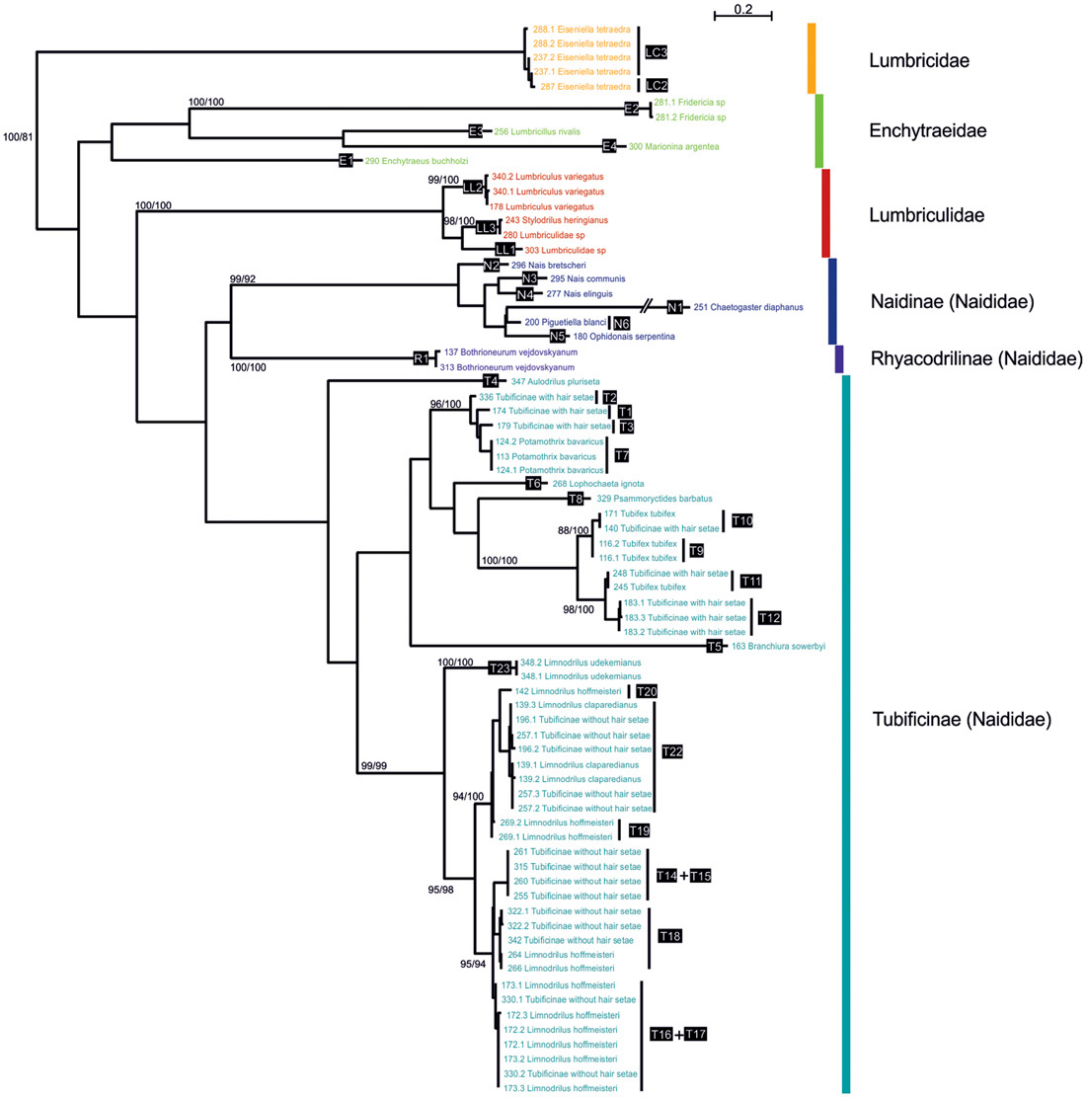
Maximum likelihood phylogenetic tree based on ITS2 sequences. The numbers indicated at the black background represent the lineage numbers reported in Table 1. The numbers above the internal nodes correspond to bootstrap values of ML and NJ analyses; only those higher than 80% are indicated. The names of taxa comprise the numbers of isolates and their clones and the morphological identification.

The topology of ITS2 tree (Fig 2), confirms the monophyly of the families, in agreement with COI data. In general, the basal branches are longer than in COI tree, suggesting high divergence between ITS2 of different subfamilies and families, with one lineage (N1) showing extremely fast evolutionary rate (Fig 2). A total of 35 lineages are distinguished, 29 of them being separated by more than 3% of sequence divergence (arbitrarily chosen threshold value). Lineages T9 and T10 (*T*. *tubifex*), lineages T18 and T16/T17 (*L*. *hoffmeisteri*) and lineages LC2 and LC3 (*E*. *tetraedra*) differ by only 1.67, 2.11 and 1.76%, respectively. The highest intra-lineage variation of lineage T22 (*L*. *claparedeanus*) is 2.12%.

The network analysis (S1 Fig) presents the patterns of variations in lineages T9-T10, T14-T15-T16-T17-T18, LC2-LC3 and T19-T20-T22. It shows that a high number of mutations (more than 10) separate lineage T9 from lineage T10, lineage T18 from lineage T16/T17 and lineage LC2 from lineage LC3. These lineages can therefore be considered as distinct. In the case of lineage T22, no sublineages were distinguished because the high intra-individual variation is due to polymorphism of ITS2 copies.

Only two cases of discrepancy between the results of COI and ITS2 analyses are observed. These cases are: the lineages T14 and T15 (Tubificinae without hair setae), separated by 18.5% with COI, but having identical ITS2 sequences (S1 Fig); and the lineages T16 and T17 (Tubificinae without hair setae and *Limnodrilus hoffmeisteri*), which are separated by 20.5% with COI, but showing no clear divergence pattern in ITS2 sequences. In the latter case, the ITS2 copies form two separate groups, but divergent sequences originate from the same specimens (isolates 173, 330) (S1 Fig). The interpretation of these two cases is additionally impeded by the fact that the specimens of the lineages T14, T15 and T16 are immature and not identifiable, it is therefore not possible to confirm with morphological analysis whether these lineages should be grouped or not.

## Discussion

The wide use of aquatic oligochaetes as bioindicators of sediment and water quality in watercourses and lakes has been hampered by the difficulties in species identification, related to the lack of distinctive morphological features and the high level of cryptic diversity. Our study demonstrates that these taxonomic issues can be surpassed by DNA barcoding.

Our data show that the morphological studies underestimate the diversity of aquatic oligochaetes inferred from genetic data. The number of genetically distinctive lineages (41) is much higher than the number of morphologically identified taxa (28) (Table 2). This number is particularly high, given a relatively low number of analysed specimens (185). In comparison, 81 taxa were identified morphologically in the same area from an assemblage of 11’650 isolated specimens [50–52].

**Table 2:** Morphological vs genetic diversity.

	Morphological approach	Genetic approach
Tubificinae	12 taxa	23 lineages
Naidinae	6 species	6 lineages
Rhyacodrilinae	1 species	1 lineage
Lumbriculidae	3 taxa	3 lineages
Enchytraeidae	4 taxa	4 lineages
Lumbricidae	2 taxa	4 lineages
Total	28 taxa	41 lineages

There are two main reasons for the underestimation of oligochaete diversity in routine monitoring studies.

At first, in aquatic environments, a large proportion of specimens is immature and cannot be identified, making most of sorted specimens unusable for diversity analysis. In our study, the majority of specimens that could not be identified morphologically are assigned to existing lineages based on their sequences, and only a few branch separately.

At second, some common morphospecies comprise a high level of cryptic diversity. The phenomenon is not new in oligochaetes and has been highlighted in many studies. Bely & Wray [25] found an important intraspecific variability in COI gene of *Nais variabilis* Piguet, 1906, *Paranais frici* Hrabe, 1941, *Paranais litoralis* (Muller, 1780) and *Stylaria lacustris* Linnaeus, 1767 and Envall et al. [21] revealed the existence of at least six lineages within *Nais communis / variabilis* complex based on the analysis of COI and 16S genes and ITS region. Our study confirms the presence of cryptic species in *Tubifex tubifex* and *Limnodrilus hoffmeisteri*, as shown previously by the analysis of mitochondrial 16S rRNA [22, 23, 53]. However, our data do not confirm the existence of cryptic lineages in *Lumbriculus variegatus* (Müller, 1774) [24] because only two specimens were examined. In *Eiseniella tetraedra*, several subspecies have been described based on morphological characters [39], but no molecular data were available. The two different lineages of this taxon reported in the present study could correspond to these subspecies.

Differences between Genbank sequences provided for *Enchytraeus buchholzi* and our sequence of this species is not unexpected as *E*. *buchholzi* is known to be a species complex [54]. The other differences between Genbank sequences and our sequences could be explained by the existence of cryptic species within *Lumbricillus rivalis*, *Aulodrilus pluriseta* and the genus *Potamothrix*.

As cryptic species can show differences in resistance to pollution, the distinction of cryptic species is particularly important for ecological studies and may help interpreting environmental conditions. Sturmbauer et al. [22] showed that the cryptic species of *Tubifex tubifex* did not have all the same resistance to Cd. *Tubifex tubifex* and *Lumbriculus variegatus* are commonly used as test organisms in ecotoxicological studies. Due to the existence of cryptic diversity within these two species, Gustafsson et al. [24] and Hallett et al. [55] recommend to systematically identify these test organisms genetically before their use in ecotoxicological studies.

As shown by our study, the distinction of cryptic species largely depends on selected DNA barcode and accepted threshold of inter-specific divergence. By using 10% threshold in COI analysis we missed four cryptic lineages distant by 6.8–9.9%. However, none of these lineages could be recognized by ITS2 data. In aquatic oligochaetes, the COI gene evolves much faster than the ITS2, with some lineages diverging by 6–25% in COI and only by 1–5% in ITS2. Our results are consistent with those of Achurra & Erséus [27] that show the existence of six *Stylodrilus heringianus* clades based on the analysis of COI that cannot be separated with ITS analysis. Nonetheless, in general, the two markers give consistent results and we observed only two cases of discrepancy between COI and ITS2 data. The analysis of other markers such as 16S rRNA and a detailed morphological study would be necessary to delimit with certainty boundaries between these lineages. However, as both contentious cases belong to the same clade of Tubificinae without hair setae, their distinction may not be essential from the biomonitoring perspective.

The application of the Automatic Barcode Gap Discovery method confirms the pertinence of the 10% threshold of COI divergence for delimitating oligochaete lineages. Concerning the discordant case (lineages LC2 and LC3), the network analysis shows a clear separation between these lineages, so we consider that LC2 and LC3 should be kept as separate lineages.

The currently used oligochaete indices are mainly based on the total species richness of oligochaetes present in the sample and on the percentages of sensitive and resistant species to toxic and organic pollution [2, 11–13, 16]. Therefore, it is imperative to select a DNA barcode that most accurately distinguishes the oligochaete species. Our comparison of COI and ITS2 shows that both molecular markers provide a good taxonomic resolution of oligochaete diversity. However, the use of COI is much more convenient from a practical point of view. PCR amplifications of COI are easy and give positive products in nearly 95% of examined specimens. Although we used the universal COI primers [42], we do not detect any contamination and direct COI sequences were always of very good quality. The ITS primers used for oligochaete identification by Envall et al. [21] and Achurra & Erséus [27] did not work for all species and the ITS2 primers we used amplified only some specimens. Moreover, the PCR products have to be cloned because of high polymorphism of ITS2 copies.

The sampling was restricted to the Geneva area as our first aim was to create a COI barcode database for aquatic oligochaetes originated from Switzerland. We observed that, for several species, our COI barcode sequences were similar to those obtained in other European countries (Sweden, Italy, Denmark) and in the North America. The scope of our work is probably not only regional but also international.

To conclude, we confirm the usefulness of COI barcode for aquatic oligochaetes and the pertinence of the 10% threshold of divergence for delimiting species. We propose to use this threshold as a standard for biomonitoring studies, as at this level of divergence the number of conflicts with other molecular markers and morphological characters is relatively limited. The creation of a comprehensive reference database is the most important challenge for more general use of oligochaetes as bioindicators of aquatic ecosystems. With further development of high-throughput sequencing technologies applied to biomonitoring [26, 56, 57], the COI-based assessment of aquatic oligochaetes diversity may become a very useful tool for ecological diagnostics.

## Supporting Information

S1 Fig Median joining network of the ITS2 sequences of the lineages T14-T15-T16-T17-T18, T9-T10, LC2-LC3 and T19-T20-T22. The numbers indicated at the black background represent the lineage numbers reported in Table 1. The areas of circles are proportional to the numbers of sequences. The numbers placed at circles corresponds to the numbers of isolates and their clones.
(TIF)

S1 Table Sampling localities. (DOC)

S2 Table List of accession numbers (European Nucleotide Archive) of COI and ITS2 sequences. (DOC)
