# Supplementary figures and images for: Molecular Barcoding of Aquatic Oligochaetes: Implications for Biomonitoring
Source: PLoS One. 2015 Apr 9;10(4):e0125485. doi: 10.1371/journal.pone.0125485 (PMC4391796; doi:10.1371/journal.pone.0125485)

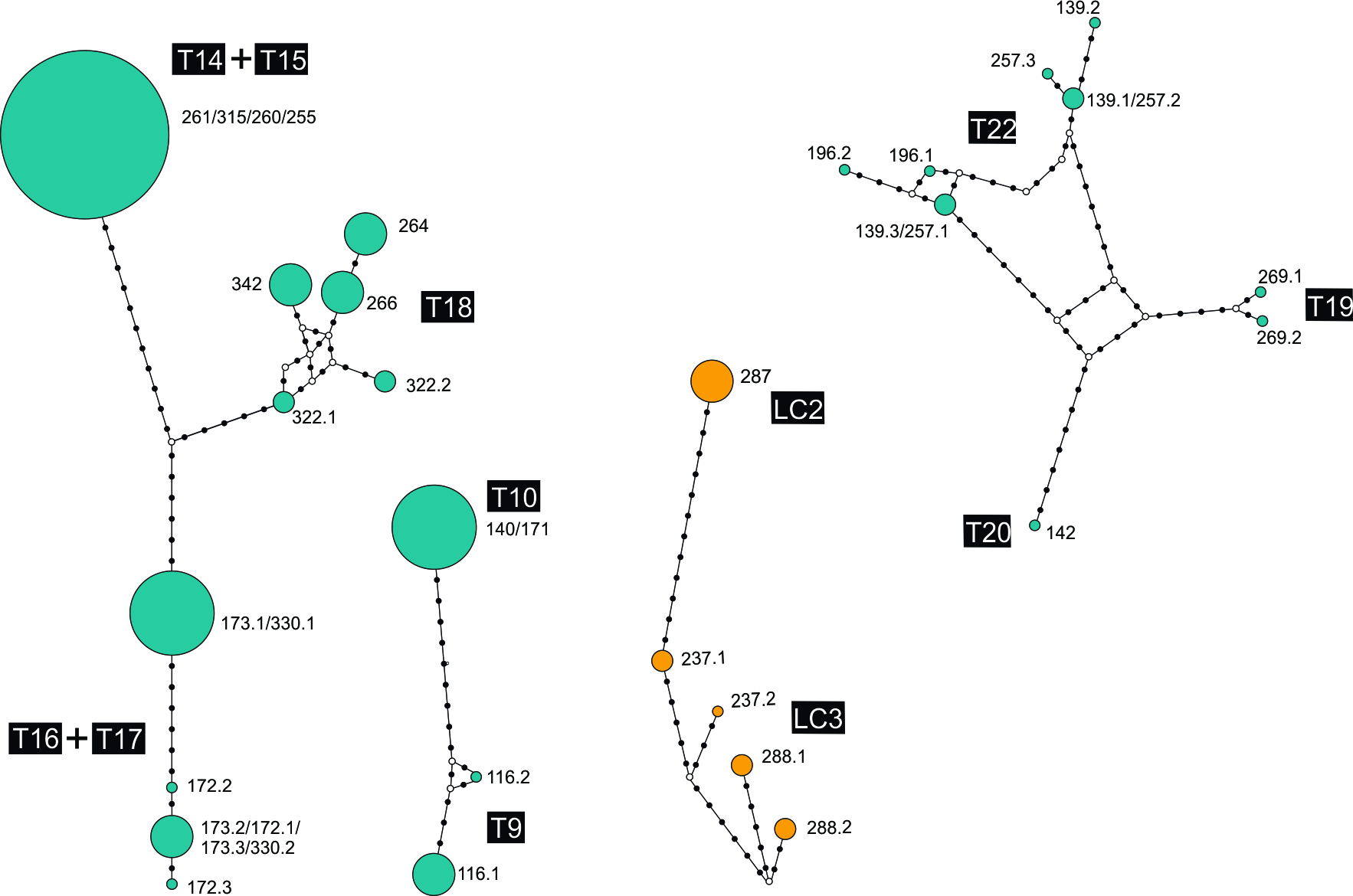

Supplement: S1 Fig — The numbers indicated at the black background represent the lineage numbers reported in Table 1. The areas of circles are proportional to the numbers of sequences. The numbers placed at circles corresponds to the numbers of isolates and their clones. (TIF) [file pone.0125485.s001.tif]
