# Supplementary material for: Molecular Barcoding of Aquatic Oligochaetes: Implications for Biomonitoring
Source: PLoS One. 2015 Apr 9;10(4):e0125485. doi: 10.1371/journal.pone.0125485 (PMC4391796; doi:10.1371/journal.pone.0125485)
Supplement: S1 Table — (DOC) [file pone.0125485.s002.doc]

**Table S1. Sampling localities**

| Watercourse | Site | X Y Coordinates | Number of sequenced specimens |
| --- | --- | --- | --- |
| Allondon | La Plaine | 46.17911°N 6.00927°E | 3 |
| Eaux-Chaudes | Passerelle aval | 46.18603°N 6.00883°E | 2 |
| Avril | Bourdigny | 46.21660°N 6.04665°E | 52 |
| Avril | Peney | 46.20488°N 6.04080°E | 7 |
| Seymaz | Amont Rouelbeau | 46.24239°N 6.21897°E | 13 |
| Seymaz | Belle-Idée | 46.20667°N 6.20157°E | 29 |
| Seymaz | De Haller | 46.20112°N 6.19612°E | 22 |
| Seymaz | Claparède | 46.18849°N 6.18508°E | 14 |
| Seymaz | embouchure | 46.18009°N 6.18203°E | 2 |
| Rhône | Jonction | 46.20199°N 6.12231°E | 2 |
| Rhône | Chèvres | 46.20271°N 6.07305°E | 9 |
| Hermance | pont de Crévy | 46.28347°N 6.24095°E | 7 |
| Crues | Aval STEP Laconnex | 46.15655°N 6.03291°E | 1 |
| Arve | Sainte-Clotilde | 46.19827°N 6.13121°E | 1 |
| stream near Chexbres |  | unknown | 16 |
| stream near Palézieux |  | unknown | 5 |
