# Supplementary material for: Molecular Barcoding of Aquatic Oligochaetes: Implications for Biomonitoring
Source: PLoS One. 2015 Apr 9;10(4):e0125485. doi: 10.1371/journal.pone.0125485 (PMC4391796; doi:10.1371/journal.pone.0125485)
Supplement: S2 Table — (DOC) [file pone.0125485.s003.doc]

**Table S2. List of accession numbers (European Nucleotide Archive) of COI and ITS2 sequences.**

|  | Lineage No | Accession No COI | Accession No ITS2 |
| --- | --- | --- | --- |
| **Naididae** |  |  |  |
| **Tubificinae** |  |  |  |
| Tub. with hair setae | T1 | LN810329 | LN810199 |
| Tub. with hair setae | T2 | LN810322 ; LN810323 ; LN810324 ; LN810325 ; LN810326 ; LN810327 | LN810201 |
| Tub. with hair setae | T3 | LN810328 | LN810200 |
| *Aulodrilus pluriseta* | T4 | LN810415 | LN810232 |
| *Branchiura sowerbyi* | T5 | LN810299 ; LN810300 ; LN810301 ; LN810302 ; LN810303 | LN810233 |
| *Lophochaeta ignota* | T6 | LN810321 | LN810205 |
| *Potamothrix bavaricus* | T7 | LN810330 ; LN810331 ; LN810332 ; LN810333 ; LN810334 ; LN810335 ; LN810336 LN810337 ; LN810338 ; LN810339 ; LN810340 ; LN810341 ; LN810342 ; LN810343 LN810344 ; LN810345 ; LN810346 ; LN810347 ; LN810348 ; LN810349 ; LN810350 LN810351 ; LN810352 ; LN810353 ; LN810354 ; LN810355 ; LN810356 ; LN810357 LN810358 ; LN810359 ; LN810360 ; LN810361 ; LN810362 ; LN810363 ; LN810364 LN810365 ; LN810366 ; LN810367 ; LN810368 ; LN810369 ; LN810370 ; LN810371 LN810372 ; LN810373 | LN810202 ; LN810203 ; LN810204 |
| *Psammoryctides barbatus* | T8 | LN810374 ; LN810375 ; LN810376 ; LN810377 | LN810206 |
| *Tubifex tubifex* | T9 | LN810419 ; LN810420 ; LN810421 ; LN810422 | LN810209 ; LN810210 |
| *Tubifex tubifex* | T10 | LN810423 ; LN810424 ; LN810425 ; LN810426 | LN810207 ; LN810208 |
| *Tubifex tubifex* | T11 | LN810378 ; LN810379 ; LN810380 ; LN810381 ; LN810382 | LN810211 ; LN810212 |
| *Tubifex tubifex* | T12 | LN810416 ; LN810417 ; LN810418 | LN810213 ; LN810214 ; LN810215 |
| *Tubifex montanus* | T13 | LN810298 |  |
| Tub. without hair setae | T14 | LN810383 | LN810185 |
| Tub. without hair setae | T15 | LN810384 ; LN810385 ; LN810386 | LN810182 ; LN810183 ; LN810184 |
| Tub. without hair setae | T16 | LN810409 | LN810170 ; LN810176 |
| *Limnodrilus hoffmeisteri* | T17 | LN810387 ; LN810388 ; LN810389 ; LN810390 ; LN810391 ; LN810392 ; LN810393 LN810394 ; LN810395 ; LN810396 ; LN810397 ; LN810398 ; LN810399 ; LN810400 LN810401 ; LN810402 ; LN810403 ; LN810404 ; LN810405 ; LN810406 ; LN810407 LN810408 | LN810169 ; LN810171 ; LN810172 LN810173 ; LN810174 ; LN810175 |
| Limnodrilus hoffmeisteri | T18 | LN810410 ; LN810411 ; LN810412 ; LN810413 ; LN810414 | LN810177 ; LN810178 ; LN810179 LN810180 ; LN810181 |
| *Limnodrilus hoffmeisteri* | T19 | LN810314 | LN810186 ; LN810187 |
| *Limnodrilus hoffmeisteri* | T20 | LN810309 ; LN810310 ; LN810311 ; LN810312 ; LN810313 | LN810188 |
| *Limnodrilus hoffmeisteri* | T21 | LN810304 ; LN810305 ; LN810306 ; LN810307 ; LN810308 |  |
| *Limnodrilus claparedianus* | T22 | LN810315 ; LN810316 ; LN810317 ; LN810318 ; LN810319 | LN810189 ; LN810190 ; LN810191 LN810192 ; LN810193 ; LN810194 LN810195 ; LN810196 |
| *Limnodrilus udekemianus* | T23 | LN810320 | LN810197 ; LN810198 |
| **Naidinae** |  |  |  |
| *Chaetogaster diaphanus* | N1 | LN810268 | LN810236 |
| *Nais bretscheri* | N2 | LN810267 | LN810237 |
| *Nais communis* | N3 | LN810253 | LN810238 |
| *Nais elinguis* | N4 | LN810258 ; LN810259 ; LN810260 ; LN810261 ; LN810262 LN810263 ; LN810264 LN810265 ; LN810266 | LN810241 |
| *Ophidonais serpentina* | N5 | LN810257 | LN810239 |
| *Piguetiella blanci* | N6 | LN810254 ; LN810255 ; LN810256 | LN810240 |
| **Rhyacodrilinae** |  |  |  |
| *Bothrioneurum vejdovskyanum* | R1 | LN810295 ; LN810296 ; LN810297 | LN810234 ; LN810235 |
| **Lumbriculidae** |  |  |  |
| Lumbriculidae unrecognizable in an immature state | LL1 | LN810271 ; LN810272 | LN810226 |
| *Lumbriculus variegatus* | LL2 | LN810269 ; LN810270 | LN810223 ; LN810224 ; LN810225 |
| *Stylodrilus heringianus* | LL3 | LN810273 ; LN810274 ; LN810275 ; LN810276 ; LN810277 ; LN810278 ; LN810279 LN810280 ; LN810281 ; LN810282 ; LN810283 ; LN810284 ; LN810285 ; LN810286 LN810287 ; LN810288 ; LN810289 ; LN810290 ; LN810291 ; LN810292 ; LN810293 LN810294 | LN810221 ; LN810222 |
| **Enchytraeidae** |  |  |  |
| *Enchytraeus buchholzi* | E1 | LN810247 | LN810231 |
| *Fridericia* sp. | E2 | LN810248 | LN810229 ; LN810230 |
| *Lumbricillus rivalis* | E3 | LN810245 ; LN810246 | LN810227 |
| *Marionina argentea* | E4 | LN810244 | LN810228 |
| **Lumbricidae** |  |  |  |
| *Dendrodrilus rubidus* | LC1 | LN810243 |  |
| *Eiseniella tetraedra* | LC2 | LN810251 ; LN810252 | LN810216 |
| *Eiseniella* tetraedra | LC3 | LN810249 ; LN810250 | LN810217 ; LN810218 ; LN810219 LN810220 |
| *Helodrilus oculatus* | LC4 | LN810242 |  |

Tub. with hair setae = unidentified immature Tubificinae with hair setae; Tub. without hair setae = unidentified immature Tubificinae without hair setae
